# Kraniale Osteomyelitis bei einer Patientin mit KID‐Syndrom: Notwendigkeit gründlicher Untersuchungen bei chronischen Wunden

**DOI:** 10.1111/ddg.15972_g

**Published:** 2026-05-05

**Authors:** Michael Wolfgang Höner, Anabelle Kainz, Cornelia Erfurt‐Berge

**Affiliations:** ^1^ Hautklinik Uniklinikum Erlangen Friedrich‐Alexander‐Universität Erlangen‐Nürnberg, Erlangen

Sehr geehrte Herausgeber,

Das Keratitis‐Ichthyose‐Taubheits‐Syndrom (keratitis–ichthyosis–deafness syndrome; KID syndrome) ist eine seltene Genodermatose mit einer geschätzten Prävalenz von weniger als 1 zu 1 000 000.^1^ Es wird hauptsächlich durch sporadische Mutationen im *GJB2*‐Gen verursacht, das für Connexin 26 kodiert, ein Protein, das für die Zell‐Zell‐Kommunikation von entscheidender Bedeutung ist.^2^ Das Syndrom ist durch eine Trias von Symptomen gekennzeichnet: Keratitis, ichthyosiforme Abschuppung (Erythrokeratoderma) und neurosensorische Taubheit. Weitere Merkmale sind Hyperkeratosen der Handflächen, Alopezie und eine Prädisposition für schwere Hautinfektionen und Malignome.^3^ Wir berichten über einen seltenen Fall eines KID‐Syndroms, bei dem eine Osteomyelitis des Schädels als Komplikation auftrat. Eine solche Konstellation ist bisher nicht beschrieben worden. Im Jahr 2023 stellte sich eine 17‐jährige Patientin mit einer bekannten KID‐Diagnose in unserem dermatologischen Wundzentrum mit refraktären Wunden am Kapillitium vor. Die Patientin wies die klassischen Merkmale des KID‐Syndroms auf: Erythrokeratodermie, Hyperkeratose, Alopezie und neurosensorische Taubheit. Genetische Tests nach Geburt bestätigten die Diagnose. Außerdem wies sie eine Kachexie (BMI 13,7), ausgedehnte okzipitale und temporale Ulzerationen mit Hypergranulation sowie frontale und parietale Abszesse auf (Abbildung 1a–f). Der Vater der Patientin hatte sich zu Hause um die Wundversorgung gekümmert und die Patientin trug regelmäßig eine Perücke, um die Alopezie und die Wunden zu verbergen. Eine Magnetresonanztomographie des Schädels (cMRT) zeigte eine chronische Osteomyelitis des Schädelknochens, die mit einem aktiven Abszess in der vorderen Kopfhaut korrespondierte. Diese Komplikation ist in der Literatur bisher nicht beschrieben worden (Abbildung 1g). Die mikrobiologische Untersuchung des Abszesses bestätigte eine Infektion mit *Staphylococcus aureus*, *Staphylococcus lugdunensis* und *Finegoldia magna*. Die Patientin wurde zur intensiven Behandlung stationär aufgenommen, einschließlich einer anfänglichen sechswöchigen antibiotischen Behandlung mit Cotrimoxazol und Metronidazol. Ein chirurgisches Débridement und eine Abszessevakuierung wurden mehrfach durchgeführt, wobei die histologische Untersuchung eine maligne Transformation ausschloss. Während der gesamten Dauer der Antibiotikatherapie bildeten sich keine neuen Abszesse. Klinisch kam es jedoch nach Absetzen der Antibiotikatherapie zu wiederkehrenden Abszessbildungen, insbesondere über zuvor infizierten Schädelbereichen (frontal, okzipital und parietal), was auf eine anhaltende Entzündungsaktivität hindeutete. Ein neurochirurgischer Eingriff wurde diskutiert, jedoch aufgrund der beeinträchtigten Wundheilung der Patientin als ungünstig bewertet und von der Patientin abgelehnt. Zudem lehnte sie einen längeren stationären Aufenthalt zur intravenösen Antibiotikatherapie ab, was die therapeutischen Optionen bei der chronischen Osteomyelitis einschränkte. Die Wundversorgung umfasste tägliche Verbandwechsel mit Polihexanid‐Lösung, silikonisierte Wundgaze bei stärker erosiven Läsionen sowie saugfähige Verbände bei stärkerer Exsudation. Der Verbandwechsel musste zunächst unter intravenöser Analgesie mit Piritramid durchgeführt werden. Nach der Entlassung wurden ein professionelles Wundmanagement und die häusliche Wundpflege fortgeführt. Das chirurgische Débridement führte zu einer deutlichen Schmerzlinderung und verbesserte die Beweglichkeit der Halswirbelsäule, die durch Narbengewebe bis zum Jugulum eingeschränkt war. Eine längere Okklusion und Reibung durch das tägliche Tragen einer Perücke könnte zu lokalen Reizungen und Infektionen beigetragen haben; Alternativen wie Kopftücher wurden von der Patientin aus psychosozialen Gründen abgelehnt. Die ausgeprägte Kachexie wurde als Folge der chronischen Infektion gewertet. Laboruntersuchungen bei der Vorstellung zeigten erhöhte Ferritinwerte (Akutphasenreaktion), verminderte Vitamin‐D‐ und Folsäurewerte, normale Vitamin‐B12‐Werte; Vitamin C und Zink wurden nicht bestimmt. Zur Behandlung der Erythrokeratodermie und Hyperkeratose wurde eine systemische Therapie mit Acitretin, einem Retinoid, mit 0,3 mg/kg Körpergewicht (KG) begonnen und im Laufe der Zeit auf 0,6 mg/kg KG erhöht. Angesichts des jungen Alters des Patienten wurden die potenziellen Nebenwirkungen einer langfristigen systemischen Retinoidtherapie – darunter Haut‐ und Schleimhauttrockenheit, Skelettveränderungen und Teratogenität – sorgfältig besprochen. Zur Gewährleistung einer sicheren Anwendung wurde eine regelmäßige labortechnische und klinische Überwachung eingeleitet. Dies führte zu einer deutlichen Verringerung des Erythrokeratodermie, wie in früheren Publikationen berichtet.^4^ Darüber hinaus wurde eine hochkalorische und proteinreiche Flüssignahrung verabreicht. Diese Maßnahmen führten insgesamt zu einer erheblichen Verbesserung der Wundheilung der Patientin, ihres klinischen Gesamtzustands und ihrer Lebensqualität. Vor dem Hintergrund der bekannten Beeinträchtigung der Wundheilung beim KID‐Syndrom ist eine gründliche diagnostische Untersuchung unerlässlich, um zusätzliche, potenziell schwerwiegende Ursachen für chronische Wunden zu erkennen, wie in diesem Fall gezeigt wurde.^5^ Zu den Präventionsstrategien bei Patienten mit KID‐Syndrom gehören eine regelmäßige klinische Überwachung, die frühzeitige Behandlung kleiner Hautläsionen, die Vermeidung von okklusiven Kopfbedeckungen und die Aufklärung der Patienten, um das Risiko schwerer Hautinfektionen zu minimieren.

**ABBILDUNG 1:**
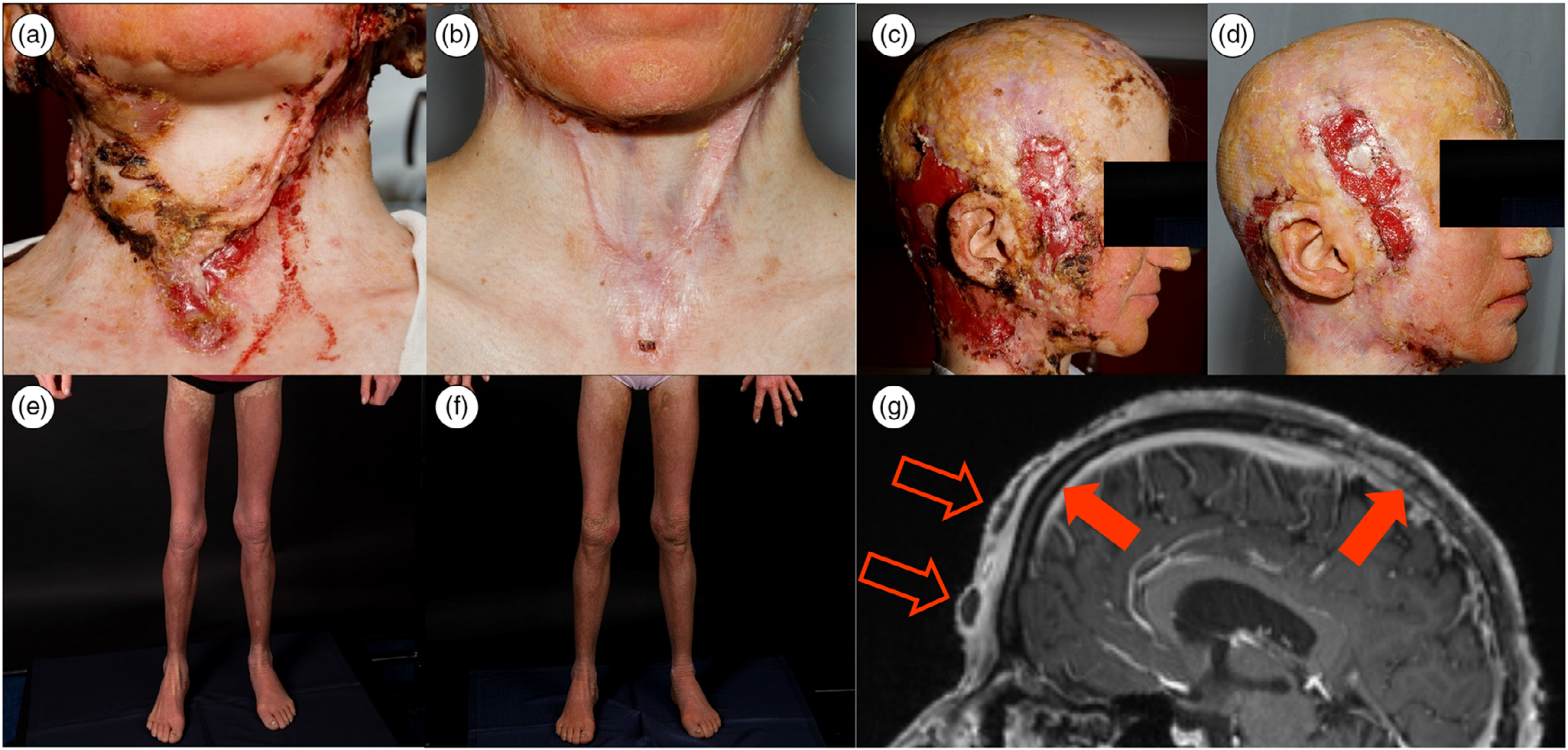
Klinisches Bild der Patientin vor (a, c, e) und nach (b, d, f) Intervention sowie (g) Befund der Magnetresonanztomographie des Schädels. (a) Multiple Hyperkeratosen, Hypergranulation und Narbenstränge in der Halsregion vor chirurgischem Eingriff und Wundversorgung. (b) Wundzustand in der Zervikalregion 9 Monate nach chirurgischem Eingriff. (c) Multiple Hyperkeratosen, Hypergranulation und Krusten in der periaurikulären Region vor chirurgischem Eingriff und Wundversorgung. Nebenbefundlich vernarbende Alopezie (d) Wundzustand in der periaurikulären Region 9 Monate nach chirurgischem Eingriff und unter intensiver Wundversorgung. (e) Erythrokeratodermie an den Beinen vor Beginn der Acitretin‐Therapie. (f) Hautzustand 6 Monate nach Beginn der Acitretin‐Therapie mit deutlichem Rückgang der Hyperkeratosen und leichter Abnahme des Erythems. (g) Sagittale T1‐gewichtete Magnetresonanztomographie nach Kontrastmittelgabe zeigt eine verstärkte Anreicherung im frontalen und parietalen Schädelbereich (gefüllte Pfeile) entsprechend einer Osteomyelitis sowie Abszessbildungen (umrandete Pfeile).

## INTERESSENKONFLIKT

Keiner.
